# Uptake and correlates of influenza vaccine among older adults residing in rural regions of south China: a cross-sectional study

**DOI:** 10.3389/fpubh.2024.1383293

**Published:** 2024-10-11

**Authors:** Peizhen Zhao, Wenqian Xu, Jinshen Wang, Peng Liang, Haiyi Li, Cheng Wang

**Affiliations:** ^1^Dermatology Hospital, Southern Medical University, Guangzhou, China; ^2^Institute for Global Health, Southern Medical University, Guangzhou, China; ^3^School of Public Health, Southern Medical University, Guangzhou, China

**Keywords:** influenza vaccine, aged, rural population, factor, China

## Abstract

**Background:**

Seasonal influenza continues to pose a substantial public health challenge for older adults residing in rural areas worldwide. Vaccination remains the most efficacious means of preventing influenza. This study aimed to investigate the extent of influenza vaccine coverage and identify the factors influencing vaccine uptake among older adults in rural regions of south China.

**Methods:**

A cross-sectional study utilizing convenience sampling was conducted in two rural sites in Guangdong Province. Individuals needed to meet specific inclusion criteria: (1) attainment of 60 years of age or older; (2) originating from rural households; (3) demonstrating a voluntary desire to partake in the survey, either through written or verbal informed consent. Data encompassed variables such as socio-demographic information, influenza infection and vaccination history, knowledge and attitudes toward influenza vaccination, and perceived beliefs regarding the influenza vaccine. Univariate and multivariable logistic regression analyses were employed to ascertain the factors associated with influenza vaccine utilization. In the multivariable model, adjustments were made for gender, age, legal marital status, highest educational attainment, and monthly income.

**Results:**

A total of 423 participants were ultimately included in this study, with the majority falling within the age range of 60–75 years (81.3%). Only one-third of the participants had received an influenza vaccine in the past year (30.0%). Notably, nearly half of the older adults exhibited hesitancy toward influenza vaccination (45.1%). The multivariable analysis revealed that rural older people with a robust understanding of influenza vaccines and a positive attitude toward them (adjusted odds ratio [aOR] = 2.60, 95% confidence interval [CI]: 1.41–4.81), along with a high level of trust in vaccination service providers (aOR = 2.58, 95% CI: 1.01–6.63), were positively associated with receiving influenza vaccination in the past year.

**Conclusion:**

This study reveals a low rate of influenza vaccine uptake among older adults residing in rural areas of south China. Given the limited adoption of influenza vaccination and the significant threat it poses, there is an urgent imperative to devise precise interventions aimed at enhancing the effectiveness of influenza vaccination programs.

## Introduction

Seasonal influenza imposes a significant global health burden, annually resulting in a substantial toll of morbidity and mortality. Recent estimates indicate an alarming prevalence of severe illness, encompassing 3–5 million cases, alongside a staggering 290,000–650,000 annual respiratory-related fatalities (1). While influenza can affect individuals across all age groups, it is imperative to recognize that older adults, aged 60 and above, face a heightened susceptibility to hospitalization and mortality upon infection (2). This vulnerability stems from a weakened immune system compounded by the presence of chronic diseases (3). A study conducted in China, utilizing data from national influenza surveillance and cause-of-death surveillance, unveiled a disconcerting annual average of 88,100 excess deaths attributed to influenza-associated respiratory diseases during the seasons spanning from 2010–2011 to 2014–2015. Strikingly, older people accounted for a significant 80% of these fatalities (4).

Immunization is the paramount strategy for influenza prevention, substantially mitigating the risk of influenza-related hospitalization among older adults by an average of 40% (5). The World Health Organization (WHO) and the technical guidelines for influenza vaccination in China (2019–2020) both endorse prioritizing individuals aged 60 and above for influenza vaccination (6). Meanwhile, a review indicated that influenza vaccination in the old adult is also cost-effective from a societal perspective (7–9). Regrettably, influenza vaccine uptake in China remains distressingly low, standing at a mere 3.8%, in stark contrast to the United States (ranging from 59.6 to 75.2%) and Europe (35.5%) (10–12). Meanwhile, a study in Florida did not find an association between influenza vaccine use among rural and urban residence (13). Significantly, the influenza vaccine is not integrated into China’s national immunization program and is available only upon request, incurring out-of-pocket expenses for vaccination, typically around $21. Exceptions to this financial burden exist in the form of local government-subsidized influenza vaccine initiatives, which are limited to select metropolitan areas (14, 15). Consequently, accessibility to vaccines may be markedly restricted for older adults residing in remote or rural regions, particularly those who possess limited knowledge about influenza and are economically disadvantaged. Paying special attention to the older adults living in rural areas is of great significance to promote health equality. Although there were studies focused on the influenza vaccine coverage among older adults, most of them did research on the developed areas (such as Beijing, Shanghai, etc.) or only focused on urban populations and lacked the analysis of the overall status of the older adults in rural China (16–19).

In light of this knowledge gap, our study aimed to scrutinize the prevalence of influenza vaccine coverage and to elucidate the influencing factors that govern vaccination uptake among older adults inhabiting rural areas of south China.

## Methods

### Study sites

Guangdong, a subtropical province in southern China, serves as a vital hub for both domestic and international trade. With a population exceeding 120 million, the province faces year-round influenza prevalence. Guangdong is composed of 21 prefecture-level cities, each with distinct economic conditions and population profiles. At its center lies the Pearl River Delta, a cluster of nine highly developed cities that contribute 80% of the province’s gross domestic product. In contrast, the other 12 municipalities, which are less developed and resource-constrained, are spread across four cities in the east, four in the west, and four in the north.

### Participants

A cross-sectional study was carried out in October 2023 at two rural sites, namely Yangcheng and Zhongluotan, both situated in Guangdong Province, China. Each site featured a single clinic that engaged in the recruitment of research participants. The choice of these clinics stemmed from their ample representation of older adults and medical personnel, including nurses and doctors, well-versed in influenza vaccination procedures. These clinics, serving as primary care facilities, catered to the healthcare needs of local residents, offering a spectrum of primary medical services. These services encompassed vaccinations, complimentary health check-ups for older people, and the dissemination of health-related information. The range of essential primary care services provided was consistent between the two clinics, encompassing the management of common medical conditions, chronic diseases, vaccination services, and other preventive public health initiatives. It is noteworthy that the majority of the study participants were regular visitors to these clinics.

To qualify for participation in the study, individuals needed to meet specific inclusion criteria: (1) attainment of 60 years of age or older; (2) originating from rural households; (3) demonstrating a voluntary desire to partake in the survey, either through written or verbal informed consent.

### Data collection

Data collection for this study employed paper questionnaires, which were crafted in consultation with the study group, influenza vaccine experts, and personnel from the local Centers for Disease Control and Prevention (CDC). In order to ensure the survey’s validity, a pilot test was administered to 10 older adults to assess the questionnaire items. It is pertinent to note that the data obtained during the pilot phase were not integrated into the final analysis. Face-to-face survey interviews were administered by local medical staff or members of the research team to all eligible participants in the clinic setting. The survey required approximately 10 min to be completed in its entirety. It is imperative to emphasize that all survey data were treated with utmost anonymity and confidentiality, with written or verbal consent being procured prior to the initiation of the survey process. To express our gratitude for their participation, participants were provided with a moisturizing cream valued at 2 United States dollars upon concluding their involvement in the study.

### Measurements

#### Social-demographic factors

The socio-demographic information gathered in this study included a range of variables such as age, gender, marital status, education, household size (including family members residing in the same home), and insurance status.

#### History of influenza infection and vaccination

The assessment of a participant’s history of influenza infection involved the use of two binary (yes/no) questions: ‘Have you experienced influenza or flu-like symptoms in the past year?’ and ‘Have you been hospitalized due to influenza or flu-like symptoms?’ The history of receiving the influenza vaccine was evaluated through the following question: ‘Did you receive the seasonal influenza vaccine in the previous year?’.

#### Knowledge and attitudes toward the influenza vaccine

The assessment of knowledge and attitudes toward the influenza vaccine involved the utilization of five items, each rated on a Likert-type scale ranging from 1 (completely agree) to 5 (completely disagree). These items covered areas related to the perceived importance, safety, and effectiveness of the vaccination. The total scores ranged from 5 to 25 with 3 categories: low (5–10), moderate (11–20), and high (21–25). The Cronbach’s *α* of knowledge and attitudes toward the influenza vaccine scale in this study was 0.869.

#### Perceived beliefs regarding the influenza vaccine

Participants’ perceived beliefs regarding the influenza vaccine encompassed several key aspects, namely their trust in healthcare providers’ guidance, self-perceived risk of infection, attitudes toward vaccination cost, and vaccine hesitancy.

Trust in healthcare providers’ advice was gauged through the employment of four items, self-perceived infection risk through four items, attitudes regarding vaccination cost through three items, and vaccine hesitancy through one item. Each of these items was rated on a Likert-type scale, spanning from 1 (completely agree/very hesitant) to 5 (completely disagree/not at all hesitant). All questionnaire items were adapted from a prior study on vaccine hesitancy among Chinese adults. Trust in healthcare providers’ advice ranged from 4 to 20 with 3 categories: low (4–12), moderate (13–15), and high (16–20). In a similar manner, self-perceived infection risk produced a total score ranging from 4 to 20, thus allowing for categorization as low (4–10), moderate (11–15), and high (16–20). Finally, attitudes toward vaccination costs generated a total score within the range of 3–15, with 3 categories: low (3–5), moderate (6–12), and high (13–15). The Cronbach’s *α* of trust in healthcare providers’ guidance scale was 0.877. The Cronbach’s *α* of self-perceived infection risk scale was 0.778. The Cronbach’s *α* of attitudes toward vaccination scale was 0.732.

### Statistical analysis

A descriptive analysis was undertaken to furnish an overview of the sociodemographic attributes and the prevalence of influenza vaccine uptake. For comparing categorical variables, the *χ*^2^ test was employed. The differences in knowledge and attitudes between the subgroup of individuals who had received the influenza vaccine and those who had not in China were assessed using the *t*-test. To scrutinize the factors linked to the utilization of the influenza vaccine, univariate and multivariable logistic regression analyses were conducted. In the multivariable model, adjustments were made for gender, age, legal marital status, highest educational attainment, and monthly income. All data analyses were executed utilizing SAS 9.2 (SAS Institute Inc., Cary, NC).

## Results

In its entirety, the survey encompassed 435 older individuals. Among this group, 9 participants were excluded due to being below 60 years of age, while 3 older people were ineligible for inclusion as they had not provided their consent. Consequently, a total of 423 (97.2%) participants were ultimately included in this study.

### Sociodemographic characteristics

Among the 423 participants, the majority fell within the age range of 60–75 years (81.3%), were predominantly female (59.1%), married (86.5%), possessed an educational background of junior high school or below (85.8%), reported a monthly income of less than 700 US dollars (83.2%), resided with their children (68.3%), held local household registrations (93.4%), and nearly two-fifths of them had chronic non-communicable diseases (42.6%). Additionally, more than half of the participants had previous knowledge of influenza (57.4%), over one-fourth had experienced influenza or flu-like symptoms in the past year (27.0%), and less than one-tenth had a history of hospitalization due to influenza or flu-like symptoms (6.6%) (Table 1).

**Table 1:** Social demographic and influenza characteristics among Chinese older, 2023 (*n* = 423).

Characteristic	Total, *n* (%)	Influenza vaccination in the past year, *n* (%)		*P*
		Yes	No	
	**423**	**127(30.0)**	**296(70.0)**	
Age (years)				
60–65	98(23.2)	12(9.4)	86(29.1)	**<0.001**
66–70	150(35.5)	57(44.9)	93(31.4)	
71–75	96(22.7)	37(29.1)	59(19.9)	
>75	79(18.7)	21(16.5)	58(19.6)	
Gender				
Male	173(40.9)	53(41.7)	120(40.5)	0.904
Female	250(59.1)	74(58.3)	176(59.5)	
Highest educational attainment				
Junior high school or below	363(85.8)	106(83.5)	257(86.8)	0.506
Senior high school or above	60(15.1)	21(16.5)	39(13.2)	
Legal marital status				
Married	366(86.5)	109(85.8)	257(86.8)	0.904
Unmarried/divorced/widowed	57(13.5)	18(14.2)	39(13.2)	
Monthly income (Dollar)				
<$150	110(26.0)	25(19.7)	85(66.9)	**<0.001**
$150 ~ 400	144(34.0)	34(26.8)	110(86.6)	
$401 ~ 700	98(23.2)	31(24.4)	67(52.8)	
>$700	71(16.8)	37(29.1)	34(26.8)	
Family situation				
Living alone	36(8.5)	10(7.9)	26(8.8)	0.373
Living with spouse	98(23.2)	35(27.6)	63(21.3)	
Living with children	289(68.3)	82(64.6)	207(69.9)	
Household registration				
Local	395(93.4)	123(96.9)	272(91.9)	0.096
Migration	28(6.6)	4(3.1)	24(8.1)	
Medical insurance				
Medical insurance for urban workers	71(16.8)	19(15.0)	52(17.6)	0.237
New Rural Cooperative Medical Care	286(67.6)	93(73.2)	193(65.2)	
Other	66(15.8)	15(11.8)	51(17.2)	
Have chronic non-communicable diseases				
Yes	180(42.6)	55(43.3)	125(42.2)	0.922
No	243(57.4)	72(56.7)	171(57.8)	
Ever heard of influenza				
Yes	243(57.4)	93(73.2)	150(50.7)	**<0.001**
No	180(42.6)	34(26.8)	146(49.3)	
Had influenza or flu-like symptoms				
Yes	114(27.0)	47(37.0)	67(22.6)	**0.002**
No	309(73.0)	80(63.0)	229(77.4)	
Ever hospitalization for influenza or flu-like symptoms				
Yes	28(6.6)	17(13.4)	11(3.7)	**<0.001**
No	395(93.4)	110(86.6)	285(96.3)	
Ever heard of influenza vaccine				
Yes	246(58.2)	116(91.3)	130(43.9)	**<0.001**
No	177(41.8)	11(8.7)	166(56.1)	

The bold values meaned “*p* < 0.05”.

### Vaccine characteristics

A mere one-third of the participants had received an influenza vaccine in the previous year (30.0%), while nearly half of the older adults exhibited hesitancy toward influenza vaccination (45.1%). Furthermore, more than half of the participants were already familiar with the concept of the influenza vaccine (58.2%). Additionally, a significant majority of the older population expressed willingness to accept free influenza vaccines donated by their peers (83.9%), as evidenced in Table 1.

The analysis revealed that participants who had received an influenza vaccination in the past year were more likely to fall within the age bracket of 66 to 75 (41.6%), have a monthly income exceeding 700 dollars (52.1%), possess prior knowledge of influenza (38.3%), have experienced influenza or flu-like symptoms in the past year (41.2%), have a history of hospitalization due to influenza or flu-like symptoms (60.7%), possess knowledge of the influenza vaccine (47.2%), exhibit a high level of influenza vaccine knowledge and positive attitudes (38.5%), exhibit a high level of influenza risk (57.1%), perceive the cost of vaccination as low (46.9%), and have no hesitations whatsoever regarding influenza vaccination (50.5%). These associations were found to be statistically significant (*p* < 0.05), as elucidated in Tables 1, 2.

**Table 2:** Influenza vaccine knowledge, attitude, vaccination service provider trust, influenza risk perception, perceived cost of vaccination, and influenza vaccination hesitancy among Chinese older, 2023 (*n* = 423).

Characteristic	Total n (%)	Influenza vaccination in the past year, *n* (%)		*P*
		Yes	No	
Influenza vaccine knowledge attitude				
Low	68(16.1)	13(10.2)	55(18.6)	**0.004**
Moderate	251(59.3)	74(58.3)	177(59.8)	
High	104(24.6)	40(31.5)	64(21.6)	
Mean ± SD	12.6 ± 3.0	13.32 ± 3.1	12.22 ± 2.9	**0.001**
Vaccination service provider trust				
Low	12(2.8)	3(2.4)	9(3.0)	0.094
Moderate	26(6.1)	3(2.4)	23(7.8)	
High	385(91.1)	121(95.3)	264(89.2)	
Mean ± SD	18.1 ± 2.3	18.66 ± 2.0	17.84 ± 2.4	**<0.001**
Influenza risk perception				
Low	201(47.5)	53(41.7)	148(50.0)	**0.006**
Moderate	194(45.9)	58(45.7)	136(45.9)	
High	28(6.6)	16(12.6)	12(4.1)	
Mean ± SD	10.9 ± 3.1	11.62 ± 3.4	10.66 ± 2.95	**0.004**
Perceived cost of vaccination				
Low	96(22.7)	45(35.4)	51(17.2)	**<0.001**
Moderate	299(70.7)	79(62.2)	220(74.3)	
High	28(6.6)	3(2.4)	25(8.4)	
Mean ± SD	7.2 ± 2.2	6.5 ± 2.1	7.5 ± 2.2	**<0.001**
Influenza vaccination hesitancy				
Very hesitant	17(4.0)	6(4.7)	11(3.7)	**<0.001**
Hesitant	68(16.1)	9(7.1)	59(19.9)	
Neutral	106(25.1)	20(15.7)	86(29.1)	
No hesitation	123(29.1)	37(29.1)	86(29.1)	
Not at all hesitant	109(25.8)	55(43.3)	54(18.2)	

Due to numerical rounding, some totals may not add up to 100%.The bold values meaned “*p* < 0.05”.

### Factors correlated with receiving the influenza vaccine

In the multivariable model, adjusted for age, marital status, highest educational attainment, annual income, and gender, several factors were found to be significantly associated with influenza vaccination in the past year. Three factors demonstrated a positive correlation with influenza vaccination uptake: possessing high influenza vaccine knowledge and a positive attitude (adjusted odds ratio [a*OR*] = 2.60, 95% confidence interval [CI]: 1.41–4.81), having a high level of trust in vaccination service providers (a*OR* = 2.58, 95% CI: 1.01–6.63) and having a high level of influenza risk perception (a*OR* = 4.36, 95% CI: 1.77–10.71).

Conversely, five factors exhibited a negative correlation with influenza vaccination in the past year: never having heard of influenza (a*OR* = 0.32, 95% CI: 0.19–0.53), not experiencing flu-like symptoms in the past year (a*OR* = 0.50, 95% CI: 0.31–0.81), no history of hospitalization due to influenza or flu-like symptoms (a*OR* = 0.25, 95% CI: 0.11–0.58), never having heard of the influenza vaccine (a*OR* = 0.05, 95% CI: 0.02–0.10), and perceiving a high cost associated with vaccination (a*OR* = 0.35, 95% CI: 0.21–0.59). Refer to Table 3 for further details.

**Table 3:** Factors correlated with influenza vaccination among Chinese older, 2023 (*n* = 423).

Characteristic	cOR (95% CI)	*P*	aOR^a^ (95% CI)	*P*
Age (years)				
61–65	Ref		–	–
66–70	4.39(2.21–8.74)	**<0.001**	–	–
71–75	4.49(2.16–9.33)	**<0.001**	–	–
>75	2.59(1.19–5.68)	**0.017**	–	–
Gender				
Male	Ref		–	–
Female	0.95(0.62–1.45)	0.819	–	–
Highest educational attainment				
Junior high school or below	Ref		–	–
Senior high school or above	1.31(0.73–2.32)	0.365	–	–
Legal marital status				
Married	Ref		–	–
Unmarried/divorced/widowed	1.09(0.6–1.99)	0.783	–	–
Monthly income (Dollar)				
<$150	Ref		–	–
$150 ~ 400	2.20(1.15–4.19)	**0.017**	–	–
$401 ~ 700	2.22(1.24–3.99)	**0.007**	–	–
>$700	2.7(1.57–4.64)	**<0.001**	–	–
Family situation				
Living alone	Ref		Ref	
Living with spouse	1.44(0.62–3.34)	0.390	1.42(0.53–3.78)	0.481
Living with children	1.03(0.48–2.23)	0.940	1.3(0.53–3.17)	0.566
Household registration				
Local	Ref		Ref	
Migration	0.37(0.13–1.08)	0.070	0.51(0.16–1.62)	0.255
Medical insurance				
Medical insurance for urban workers	Ref		Ref	
New rural cooperative medical Care	1.32(0.74–2.36)	0.350	0.96(0.48–1.92)	0.915
Other	0.8(0.37–1.75)	0.585	0.83(0.36–1.93)	0.664
Have chronic non-communicable diseases				
Yes	Ref		Ref	
No	0.96(0.63–1.46)	0.837	1.22(0.78–1.91)	0.391
Ever heard of influenza				
Yes	Ref		Ref	
No	0.38(0.24–0.59)	**<0.001**	0.32(0.19–0.53)	**<0.001**
Had the flu or flu-like symptoms in the past year				
Yes	Ref		Ref	
No	0.50(0.32–0.78)	**0.002**	0.50(0.31–0.81)	**0.005**
Ever hospitalization for influenza or flu-like symptoms				
Yes	Ref		Ref	
No	0.25(0.11–0.55)	**0.001**	0.25(0.11–0.58)	**0.001**
Ever heard of influenza vaccine				
Yes	Ref		Ref	
No	0.07(0.04–0.14)	<0.001	0.05(0.02–0.10)	**<0.001**
Influenza vaccination hesitancy				
Very hesitant	Ref		Ref	
Hesitant	0.28(0.08–0.94)	**0.040**	0.26(0.07–0.92)	**0.037**
Neutral	0.43(0.14–1.29)	0.131	0.41(0.13–1.29)	0.127
No hesitation	0.79(0.27–2.29)	0.663	0.79(0.26–2.41)	0.674
Not at all hesitant	1.87(0.64–5.41)	0.250	1.71(0.55–5.28)	0.353
Influenza vaccine knowledge attitude				
Low	Ref		Ref	
Moderate	2.48(1.39–4.46)	**0.002**	2.72(1.47–5.04)	**0.001**
High	2.37(1.34–4.20)	**0.003**	2.60(1.41–4.81)	**0.002**
Vaccination service provider trust				
Low and moderate	Ref		Ref	
High	2.44(0.99–6.00)	0.051	2.58(1.01–6.63)	**0.049**
Influenza risk perception				
Low	Ref		Ref	
Moderate	1.19(0.77–1.85)	0.436	1.31(0.81–2.12)	0.270
High	3.72(1.65–8.38)	**0.001**	4.36(1.77–10.71)	**0.001**
Perceived cost of vaccination				
Low	Ref		Ref	
Moderate and high	0.39(0.24–0.62)	**<0.001**	0.35(0.21–0.59)	**<0.001**

^aAdjusted for gender, age, legal marital status, highest educational attainment, and monthly income.The bold values meaned “p < 0.05”.^

## Discussion

Influenza remains a formidable global public health challenge. The most effective preventive measure against influenza is vaccination, with the older people being prioritized for such vaccination. Studies have indicated that influenza vaccination for the old adult is cost-effective (7–9). Our study reveals a notable deficiency in influenza vaccine coverage among older people in rural China. By focusing on this demographic, monitoring prevalence, and probing into the associated factors affecting vaccine coverage, our study contributes to the existing body of literature. The insights gleaned from this study hold the potential to bolster vaccination rates among the older people in China.

Our findings indicate a low rate of influenza vaccine uptake among rural older people in China, a trend that aligns with similar observations in previous Chinese studies, but is lower than vaccination rates in the United States and Canada (12, 20). However, the rate is higher than the COVID-19 vaccination rate in most high-income countries from a scoping review about COVID-19 vaccine hesitancy (21). Several factors may underlie this suboptimal vaccination rate. First and foremost, rural older people exhibit limited awareness of the influenza vaccine. Such low awareness, coupled with perceived disease risk, serves as a deterrent to vaccination. Additionally, older adults in rural areas of China encounter significant challenges in accessing influenza vaccination services, including geographic barriers, limited healthcare resources (22). Finally, the variation in vaccination programs and public health policy might contribute to the differences in vaccination rates. A review also clearly indicated gaps for evidence on system-based level or political strategies to improve vaccination uptake among older adults (23). Currently, only a few areas with budget surpluses in China are trying to integrate influenza vaccination into government financial subsidies or medical insurance reimbursement schemes, providing free vaccination for the older people. In contrast, in most regions, the older people must bear the cost of vaccination. Given the high cost-effectiveness of influenza vaccination for the old adult (7), developed countries such as the United States, the United Kingdom, and Australia have already incorporated influenza vaccination into national immunization programs, offering free vaccines to the older people (12, 24, 25). Considering the low coverage of influenza vaccination among the older people and the serious health threats posed by influenza, China urgently needs to devise effective strategies to promote vaccine uptake for older adults, such as expanding national immunization programs to include free vaccinations, establishing mobile vaccination units to reach older adults who have limited access to healthcare facilities in rural areas.

We found that approximately three-quarters of the rural older people exhibits a moderate and high level of perceived cost of vaccination. This may be primarily attributed to lower income levels among older people in rural areas (26). Our study found that approximately one-quarter of older people in rural areas have a monthly income below $150. However, previous studies and our findings have shown that older people with a higher level of perceived cost of vaccination are less likely to be vaccinated (26, 27). In the context of influenza vaccines being currently categorized as self-paid vaccines in China, many studies are exploring novel economic interventions to promote vaccine uptake among older people, such “pay it forward” initiatives (16, 28, 29), offering financial or social incentives to encourage older adults to get vaccinated and local free government influenza vaccination policy. Additionally, previous studies also suggest that launching public awareness campaigns to highlight the long-term cost benefits of flu vaccination, emphasizing potential savings on medical expenses, sick days, and productivity losses, can mitigate perceived cost risks associated with vaccination (30, 31). Hence, developing national-level public health campaigns to raise awareness about the benefits of influenza vaccination is necessary for older populations who are at higher risk of complications.

We found a low level of knowledge, attitude, and risk perception regarding influenza vaccine among older people, only one quarter of the older people possesses a high level of knowledge, attitude, and risk perception. We also found that older people with heightened knowledge and a greater perception of the risk associated with flu vaccines exhibit higher vaccination rates, which was consistent with other studies (32, 33). This finding was also similar with other studies about COVID-19, SARS and MERS pandemics (34). This phenomenon is primarily attributable to the fact that increased knowledge and heightened risk perception empower individuals to make proactive decisions to protect themselves and their communities against influenza, consequently leading to increased vaccination rates. Given the low level of influenza vaccine knowledge, it is imperative to initiate public health campaigns aimed at educating the older people on the significance of vaccinations and the potential risks associated with abstaining from vaccination. A combination of traditional and digital media channels, including television, radio, newspapers, social media, and online platforms, can be leveraged to reach a wide-ranging audience. Collaborations with local healthcare providers, community leaders, and influential figures can further facilitate the dissemination of information regarding vaccination’s importance (35).

It is important to acknowledge the limitations of our study. First, the data regarding influenza vaccine uptake were gathered via voluntary self-reporting, introducing the potential for information bias. Second, as this was a cross-sectional study, the observed correlations between influenza vaccine uptake and knowledge and attitudes should be interpreted as associations rather than causation. Third, while our study examined older adults in rural areas, the findings may not necessarily extrapolate to older adults in urban settings in China or those residing outside of China.

## Conclusion

In this study, we found a low influenza vaccine uptake among older people residing in rural China. Notably, influenza vaccine utilization is positively associated with factors such as knowledge about influenza, positive attitudes toward vaccination, and an increased perception of the risks associated with the influenza virus. In light of the suboptimal vaccination rate and the considerable health risks posed by influenza, it is crucial to devise targeted interventions aimed at augmenting influenza vaccine coverage.

## Data Availability

The raw data supporting the conclusions of this article will be made available by the authors, without undue reservation.
